# Losing Money and Motivation: Effects of Loss Incentives on Motivation and Metacognition in Younger and Older Adults

**DOI:** 10.3389/fpsyg.2020.01489

**Published:** 2020-07-15

**Authors:** Hyesue Jang, Ziyong Lin, Cindy Lustig

**Affiliations:** ^1^Department of Psychology, University of Michigan, Ann Arbor, MI, United States; ^2^Max Planck Institute for Human Development, Center for Lifespan Psychology, Berlin, Germany

**Keywords:** loss incentives, working memory, motivation, metacognition, cognitive aging

## Abstract

Incentives are usually expected to increase motivation and cognitive control and to thereby improve performance. A small but growing number of studies have begun to investigate whether the effects of incentive on cognitive performance differ for younger vs. older adults. Most have used attention and cognitive control paradigms, trial-wise implementation of incentive condition, and gain incentives (reward), with only a very few investigating the effects of loss incentives. The present study takes a complementary approach: We tested younger and older adults in a working memory paradigm with loss incentives implemented session-wide (between subjects). We also included self-report measures to ask how loss incentive affected participants’ perceptions of the mental demand of the task, as well as their perceived effort, frustration, motivation, distraction, and metacognitive judgments of how well they had performed. This allowed us to test the disparate predictions of different theoretical views: the intuitive hypothesis that incentive should increase motivation and performance, the motivational shift proposal that older adults are especially motivated to avoid losses (Freund and Ebner, 2005), a heuristic “positivity effect” perspective that older adults ignore losses (Brassen et al., 2012; Williams et al., 2017), and a more nuanced view that suggests that when negative information is unavoidable and increases perceived costs, older adults may instead disengage from the situation (Charles, 2010; Hess, 2014). The results seemed most consistent with the more nuanced view of the positivity effect. While neither group showed incentive-related performance differences, both younger and older adults reported greater perceived demand and frustration under loss incentive, especially in the most challenging conditions. Loss incentive increased the accuracy of immediate metacognitive judgments, but reduced the accuracy of later, more global judgments of competency for older adults. Self-report measures suggested that the loss incentive manipulation was distracting to young adults and demotivating for older adults. The results suggest a need for caution in generalizing from existing studies to everyday life, and that additional studies parameterizing critical aspects of task design and incentive manipulation are needed to fully understand how incentives affect cognition and motivation in younger and older adults.

## Introduction

The enthusiasm for this Research Topic in Frontiers reflects the rising interest in the last 10 years on the effects of monetary incentives on cognition. That interest was sparked in part by the integration of cognitive and computational perspectives on reinforcement learning and has spread to the effects of incentive on other aspects of cognition. The general assumption is that incentives increase motivation and that motivation in turn increases the engagement of attention and cognitive control (Botvinick and Braver, 2015; Yee and Braver, 2018). As the papers in this issue, as well as several recent reviews, indicate, a great deal of progress has been made on this topic in a relatively short period of time. However, several important gaps in the literature remain.

First, most studies have built on the reinforcement learning literature and implemented incentives on a within-subjects, trial-wise basis (i.e., comparing performance on rewarded vs. unrewarded trials). A common finding in that literature is that older adults show reduced neural responsivity to anticipated losses but similar results to young adults for anticipated gains, experienced gains, and experienced losses (reviewed by Samanez-Larkin and Knutson, 2015). Trial-wise incentive manipulations likely translate well to real-world reinforcement learning and value-based decision-making (e.g., after repeated exposures, one learns that Restaurant A is more likely to produce a rewarding experience than Restaurant B). However, in these cases, as well as in studies examining the prioritization of high- vs. low-value items in episodic memory (Castel et al., 2002; Cohen et al., 2016), incentive valence and magnitude attach to specific items, actions, or decision options.

It is not clear that conclusions from these more specific, trial-wise incentive manipulations apply to most “real world” (e.g., school, work, or sports) situations with incentivized performance. For example, a junior accountant performing an audit would likely receive bonus pay for completing all the steps needed thoroughly and efficiently (or have their pay docked for underperforming), rather than having one step be associated with bonus pay for correct completion and another step associated with lost pay for failure (e.g., Libby and Lipe, 1992). The same is likely true in many cognitively challenging situations in everyday life: following directions to reach a desired location, debugging a computer program, or organizing a weekly work schedule for oneself or a group of employees.

Second, many of these real-world situations rely heavily on working memory, and age differences in working memory are both large and a topic of central interest in both theoretical work and empirical studies of cognition and performance (see Park and Festini, 2017 for a recent review). However, most performance-incentive studies have focused on measures related to attention and cognitive control (Di Rosa et al., 2015; Schmitt et al., 2015, 2017; Williams et al., 2017, 2018; Yee et al., 2019), and only a handful have compared young and older adults. As noted above, there have also been a number of reinforcement learning and episodic memory studies focusing more on the ability to learn reward/loss associations or prioritize high vs. low reward items (e.g., Castel et al., 2002; Cohen et al., 2016), as well as studies on incentivized episodic memory encoding (e.g., Spaniol et al., 2014; Geddes et al., 2018).

To our knowledge, only one study has examined the effects of incentive on working memory in both younger and older adults (Thurm et al., 2018). The lack of studies on how incentives might affect working memory performance in younger and older adults stands in contrast to the training and neurostimulation literatures, where working memory is a frequent target because of its large age differences and importance in everyday life (Basak et al., 2008; Buschkuehl et al., 2008; Li et al., 2008; Stephens and Berryhill, 2016; Rhodes and Katz, 2017; Di Rosa et al., 2019). From a scientific perspective, another reason to examine working memory is that the range of set sizes used in many working memory tasks also provides a relatively straightforward way of examining whether age differences in the response to incentive vary as a function of task load.

Third, many studies have focused on reward (“gain”) incentives (e.g., Castel et al., 2002; Spaniol et al., 2014; Cohen et al., 2016; Thurm et al., 2018; Di Rosa et al., 2019; Yee et al., 2019; Bowen et al., 2020). However, loss is thought to play an increasingly important part in older adults’ experience, and real-world attempts to motivate their behavior often focus on the opportunity to avoid such losses (e.g., of health, of employment or financial stability, of driving privileges). Finally, the assumption that incentive will increase motivation (and then increase attention and control) is rarely tested directly. This is despite an earlier literature – interestingly, often in more ecologically valid settings – indicating that extrinsic motivators such as monetary incentive can often have paradoxical effects (see meta-analytic reviews by Deci et al., 1999; Cerasoli et al., 2014).

The present study begins to address some of these gaps. We examined the effects of loss incentive, implemented across the entire session, on both younger and older adults. We examined both working memory performance and subjective reports of related constructs including perceived demand, frustration, motivation, distraction, and metacognition. We focused on losses both because they have been understudied and because different theoretical perspectives make competing hypotheses about the effects of loss incentives on older adults, whereas predictions are the same (and thus the incentive manipulation less incisive) for reward (“gain”) effects. The subjective measures were used to provide potentially converging or disconfirming evidence for each of these views.

Before describing the rationale for our study, we review different theoretical perspectives that make disparate predictions for the effects of loss on older adults’ cognitive performance and subjective response. See Analyses for a summary of the major predictions of each view, and how they will be assessed in the current study.

First, *the intuitive prediction* is that incentive increases motivation, which increases performance. This might also be expected to reduce perceived demand and increase metacognitive accuracy, as participants pay closer attention to their performance in order to improve it. Building off of lifespan development theory and the idea that losses become more prominent in later life, the *motivational shift* hypothesis is that older adults are particularly motivated to avoid losses: “With advancing age, however, personal goals are expected to shift toward an increasingly stronger focus on maintenance and prevention of loss” (Freund and Ebner, 2005). If one follows the logical chain, described above – that greater motivation should increase the application of cognitive control and thus increase performance – this hypothesis would seem to suggest that older adults would show even larger performance and motivation increases in the loss condition than do young adults.

However, the motivational shift theory appears to primarily apply to older adults’ goal setting and preferences in decision-making scenarios, and in particular whether one gravitates toward opportunities for growth and improvement in cognitive or physical performance vs. maintenance or compensation for loss on those fronts (e.g., Freund and Ebner, 2005; Best and Freund, 2018). It may also be of relevance in avoidance-learning paradigms, where older adults have sometimes shown faster learning in response to loss (Frank and Kong, 2008; Eppinger and Kray, 2011; Hämmerer et al., 2011). It does not seem to straightforwardly apply to the motivation-cognitive performance questions of interest here. Indeed, those studies that have examined the effects of loss incentive on older adults’ response to cognitive demands are relatively consistent in showing that older adults have either an equivalent or reduced response to loss incentive compared to young adults and/or to positive incentive (e.g., Bagurdes et al., 2008; Di Rosa et al., 2015; Schmitt et al., 2015, 2017; Pachur et al., 2017; Williams et al., 2017, 2018). Thus, while we note that the motivational shift hypothesis might superficially appear to predict larger performance improvements, greater motivation, and increased metacognitive accuracy for older adults in the loss condition, we do not consider it likely to apply to the current study.

Most of the studies finding apparently reduced sensitivity to loss incentives in older adults have interpreted it as an example of the *positivity effect* – the finding that older adults tend to prioritize positive, and deprioritize negative, information for attention and memory (Bagurdes et al., 2008; Di Rosa et al., 2015; Pachur et al., 2017; Williams et al., 2017, 2018). This interpretation of the positivity effect would seem to predict that, compared to young adults, older adults should show less effects of loss incentive (results more similar to the control condition) on both our performance and subjective measures.

However, some caution is needed in making that leap. As noted above, in some situations, older adults are in fact even more responsive to loss than are young adults (Frank and Kong, 2008; Eppinger and Kray, 2011; Hämmerer et al., 2011). The apparent reduction in sensitivity to loss in some other studies may be at least partially an artifact of how incentive cues were implemented in those experiments. In most cases, the reduced loss sensitivity of older adults primarily concerns neural or electrophysiological responses to the incentive cue. Overall performance quality often shows similar incentive effects for the two age groups, although there may be some differences in speed–accuracy tradeoffs (e.g., Schmitt et al., 2015; Williams et al., 2017, 2018). This suggests that older adults may be less responsive to loss-incentive cues, but equally (and in some cases, even more so) responsive to the actual delivery of loss incentive. That interpretation would fit with findings from the reinforcement learning literature that older adults have reduced neural and arousal responses to loss cues but equivalent or greater responses to loss delivery [reviewed by Samanez-Larkin et al. (2007)].

Similar results indicating potentially greater responses by older adults to loss delivery have been reported in the Monetary Incentive Delay task (Kircanski et al., 2018). In addition, using an analysis approach that emphasizes spatiotemporal covariance patterns, Spaniol et al. (2015) found that at cue presentation, young and older adults showed similar reward-network recruitment, but older adults showed increased recruitment of frontal–parietal control networks and decreased deactivation of the default network; these effects did not differ by valence. At the point of feedback/incentive delivery, young and older adults again showed similar patterns related to general feedback/reward processing, but older adults recruited two additional networks in response to error feedback and to loss (Bowen et al., 2019).

A neuroimaging study by Geddes et al. (2018) generally replicated the pattern of a specific reduction in older adult’s activation of reward networks in response to loss cues for the Monetary Incentive Delay task but a different pattern for incentivized encoding trials for an upcoming (24 h delay) recognition memory test. Behaviorally, young adults showed incentive (reward or punishment) advantages on recollection but not familiarity; older adults had low recollection performance and no effects of incentive (see Spaniol et al., 2014 for slightly different results as well as the Geddes et al. discussion of the similarities and differences between these studies). Interestingly, the neuroimaging data showed similar activations of memory- and reward-related region in both young and older adults during the incentive cue, regardless of incentive valance, but reduced engagement of these regions by older adults during the encoding period. The authors suggest that differences between their memory task vs. the Monetary Incentive Delay task as well as value-directed memory tasks in terms of the immediacy of feedback/incentive manipulation – and thus the ability to modulate processing in response – might partially explain the differences in results.

In short, whether older adults show the same, less, or more responsivity to loss than do young adults seems to vary widely across different paradigms. A more nuanced view of the positivity effect, integrated with the concepts of proactive vs. reactive control, may provide a more comprehensive explanation for the patterns seen across different tasks. Both theoretical and empirical work indicate that the age-related positivity effect is primarily seen in low-constraint situations that allow or require older adults to direct their attention toward or away from emotional information (see Reed and Carstensen, 2012; Carstensen and DeLiema, 2018 for reviews). It does not usually occur when negative information is highly salient or otherwise processed relatively automatically. Likewise, the Dual Mechanisms of Control theory’s perspective on aging is that older adults are less likely than young adults to engage self-initiated proactive control to prepare for upcoming cognitive demands but often show even greater (perhaps compensatory) reactive control when the critical stimulus is presented (Braver, 2012; see earlier work by Craik and Byrd, 1982, for similar ideas on age differences in self-initiated processing). Thus, in many previous studies using trial-by-trial incentive cues, older adults may have failed to engage with the loss cues at presentation. This could explain the failure to show the same neural or physiological responses to those cues as did young adults. Notably, one study using block-wise presentation of incentive cues found if anything increased sensitivity to loss cues in older adults, suggesting that experienced (rather than merely anticipated) losses carried over to subsequent trials (Schmitt et al., 2017).

It has been suggested that when negative information is unavoidable, older adults may instead disengage or distance themselves from the situation and, in addition, may later reframe the situation to take a more positive view (Charles, 2010). For example, Charles and Carstensen (2008) found that after participants listened to conversations ostensibly consisting of disparaging remarks about them, young adults wanted to learn more about the cause of the complaints and made more appraisals about the speakers, whereas older adults distanced themselves from the situation with remarks such as “you can’t please all the people all the time.” Compared to incentive cues, the actual delivery of loss feedback – especially performance-based incentives in a domain (memory) that is important to older adults (Reese et al., 1999; Dark-Freudeman et al., 2006) – may be more personally relevant and thus difficult to ignore and paradoxically lead older adults to disengage from the situation rather than increase their motivation to improve (but see Barber and Mather, 2013; Barber et al., 2015, for evidence suggesting a non-linear relationship).

A related proposal from Selective Engagement Theory (SET; Hess, 2014) is that a person’s motivation to engage depends on their calculation of benefits vs. costs of that engagement, and that those costs – and thus the likelihood of disengagement – may occur at earlier levels of objective task difficulty for older adults. Although to our knowledge Hess and colleagues have not directly addressed the question of monetary incentives, if losses after error incentives magnify the perceived costs of performance, they would be predicted to increase the likelihood of disengagement. Consistent with this idea, previous studies in our lab using an attention task found that loss incentives reduced focused-attention performance and increased self-reported mind wandering in older adults (Lin, 2018; Lin et al., 2019).

An alternative, more “competitive” pathway to disengagement has been suggested by Ferdinand and Czernochowski (2018): Processing incentive information may itself create a cognitive load that draws cognitive processing away from the task. Thus, incentive could paradoxically reduce performance, with effects presumably most evident at the highest working memory loads. Alternatively, as suggested in some of their papers, the cognitive load of the task may cause older adults to ignore or less completely process incentive information (Schmitt et al., 2015, 2017). Thus, the predictions that this view would make for many of the measures in the current study are not entirely clear. As a first step toward testing this possibility, we asked participants about the degree to which they found the feedback (control or incentive) provided to them to be distracting.

## Materials and Methods

### Rationale and Overview of Methods for the Present Study

As noted earlier, although the number is small, there have been several studies examining age differences in the response to loss incentives on cognitive control tasks using the trial-based incentive cue method borrowed from reinforcement learning paradigms. These have generally indicated a reduced responsivity to loss cues in older adults, although that reduced responsivity is typically most evident on neural or physiological measures, rather than performance. Although these studies are interesting and important, it was not our goal to add another variation.

Instead, our aim was to take a first step toward closely related questions that have been thus far largely unaddressed. We used a session-wide incentive manipulation rather than trial-wise changes, since as noted above, session-wide incentives are more likely to reflect real-world situations. We examined working memory, which thus far has been the focus of only one age × incentive study despite the importance of working memory to cognitive performance in many domains and its well-known decline in aging. We focused on losses, rather than gains, since this again has been a neglected area despite the putatively increased importance of loss in later adult life, and because most of the theoretical perspectives above have the same predictions for rewards/gains but differ in their predictions for losses, making the latter more incisive.

Based in part on other data from our lab suggesting that loss incentive reduced focused attention in older adults and increased mind wandering (Lin et al., 2019), we were especially interested in the possibility that loss incentive might lead older adults to disengage from the task. Our task and procedures thus closely followed those previously used by Hess et al. (2016) to examine age differences in a physiological measure of task engagement as a function of working memory load. We used largely the same working memory task and questionnaires to assess self-reported mental demand, effort, and related constructs such as frustration, and added the loss-incentive manipulation. This also allowed our control sample to provide a basic replication test of the behavioral age differences reported by Hess et al., 2016. Finally, we added an exploratory set of subjective measures of motivation, distraction, and metacognition as a first step toward examining the effects of loss incentives on these constructs in young and older adults.

### Participants

Eighty-five young adults and 84 older adults were included in the analysis (Table 1; see Supplementary Material S8 for exclusion information). Young adults (61 female, mean age = 19.99 years, range = 18–29) were students recruited from the University of Michigan. Older adults (52 female, mean age = 71.67, range = 60–88) were recruited from the Ann Arbor community. Participants were screened to ensure physical and psychological health with no history of anxiety, depression, attention deficit hyperactivity disorder (ADHD), or head injury, and no use of medications that could affect cognition. As in other studies in our lab, the Extended Range Vocabulary Test Version 3 (ERVT; Ekstrom, 1976) was used to screen for participants who might not understand the instructions or were generally unmotivated or not willing/able to complete the experimental session; a minimum score of 9 out of a possible 48 was required. For older adults, a Mini-Mental State Examination score (MMSE; Folstein et al., 1983) of 27 or greater was required. Young and older adults received $10 and $12 per hour, respectively, for their participation (older adults received a slightly higher amount to compensate for their driving to the testing site). Written informed consent was obtained from all participants. The study was approved by the Institutional Review Board (IRB) of the University of Michigan.

**TABLE 1:** Demographics and self-reported Poor Attentional Control (PAC).

	**Young control (*n* = 43, 31 f)**	**Young loss (*n* = 42, 30 f)**	**Old control (*n* = 41, 24 f)**	**Old loss (*n* = 43, 28 f)**
**Age**				
Mean	20.19	19.79	71.37	71.95
*SD*	1.93	2.06	6.83	6.39
**Years of education**				
Mean	14.40	14.04	17.45	17.21
*SD*	1.53	1.42	2.11	2.30
ERVT				
Mean	19.65	17.95	29.51	30.33
*SD*	5.88	4.73	9.04	8.41
**PAC mind wandering**				
Mean	14.58	15.86	12.15	12.47
*SD*	4.29	3.06	3.06	3.06
**PAC boredom**				
Mean	13.72	14.81	10.51	10.79
*SD*	3.51	3.37	2.66	2.72
**PAC distractibility**				
Mean	15.42	15.67	12.39	13.79
*SD*	3.53	4.18	3.12	3.94
**MMSE**				
Mean	n/a	n/a	28.83	28.95
*SD*	n/a	n/a	1.18	1.11

**f, female; ERVT, The Extended Range Vocabulary Test; PAC, the Poor Attentional Control scale.**

### Design

Age group (young, old) and incentive condition (control, loss) were the group-level, between-subjects variables; set size was a within-subjects variable of secondary interest. Participants within each age group were randomly assigned to the control or loss condition. Our previous study using an attention task (Lin et al., 2019) found an effect size of *f* = 0.217 (equivalent $\eta_{\text{p}}^{2}$ = 0.045) for the age (young vs. old) by motivation (control vs. loss) interaction on task performance. Power analysis using *G^∗^Power* (Faul et al., 2007) suggested a total sample size of 169 to detect the age by motivation interaction with an effect size *f* of 0.217; α error probability of 0.05; power (1 - β probability) of 0.80; numerator degrees of freedom of 1; four groups in a two-way ANOVA. For the exploratory correlation analyses within each group, a sensitivity analysis indicated that *r* of 0.304 was the minimum to be detected at 0.80 power.

### Working Memory Task

The Letter Number Sequencing (LNS) task from the Wechsler Adult Intelligence Scale-III (Wechsler, 1997) was used to measure working memory. The task was programmed using PsychoPy version 3 (Peirce, 2007) and presented on a Dell PC computer. On each trial, participants received intermixed letters and numbers at a rate of one item per second. Participants were asked to report the numbers in numerical order, the letters in alphabetical order. Each run had six trials of the same set size (the number of items to be memorized). Set size increased in an ascending order across runs, from set size 2 (run 1) to set size 9 (run 8). There were eight runs in total. At the end of each run, participants were given performance feedback (percent correct/incorrect for a given run). For interactions with the within-subjects variable set size, sensitivity analyses indicated power of 0.80 for *f* = 0.111, which is equivalent to $\eta_{\text{p}}^{2}$ = 0.012 (4 groups, 8 measures, *r* = 0.217 between measures; non-sphericity correction set at 1).

### Questionnaires

All questionnaires were self-administered after the instructions for it were provided by the experimenter and the participant given the chance to ask any questions.

#### Poor Attentional Control Scale

The Poor Attentional Control (PAC) scale serves as a trait measure of attentional function in everyday life. It was administered before the LNS task to avoid the possibility that participants’ perceptions of their performance might influence their responses. The PAC subscale consists of 15 items identified by factor analysis (Huba et al., 1982) from the larger 36-item Imaginal Processes Inventory (Singer and Antrobus, 1970). As in previous studies in our lab (e.g., Berry et al., 2014a, b; Kim et al., 2017), participants completed all 36 items so that they were viewed in context, with analyses focused on the PAC scale items. For each item, the participant indicated how true the statement was for them (1 = *not all true of me*; 5 = *very true of me*).

#### NASA Task Load Index

The NASA Task Load Index (NASA-TLX) measures subjective workload experienced during the task (Hart and Staveland, 1988). It was administered after each LNS run, and it has six subscales that ask the following: (1) How mentally demanding was the task? (Mental Demand); (2) how physically demanding was the task? (Physical Demand); (3) how hurried or rushed was the pace of the task? (Temporal Demand); (4) How successful were you in accomplishing what you were asked to do? (Performance); (5) How hard did you have to work to accomplish your level of performance? (Effort); (6) How insecure, discouraged, irritated, stressed, and annoyed were you? (Frustration). The responses are rated on a 0 (very low) to 100 (very high) point scale, except for the Performance scale, which uses a “reversed” scale, 0 (successful) to 100 (failure). In the results and figures below, we present the results for the Performance scale using the more intuitive 0 (failure), 100 (success) format.

#### State Attention and Motivation Questionnaire

The State Attention and Motivation Questionnaire (SAMQ) was administered after finishing the LNS task and the final NASA-TLX form. It was created by our lab to ask “state” questions related to boredom, difficulty focusing attention, distraction, and motivation using the same wording as the “trait” level PAC scale. It has been shown in several previous studies to correlate with both the PAC trait measures and with construct-related performance measures (e.g., Berry et al., 2014a, b; Kim et al., 2017). The version used in the present study modified the last two questions to specifically assess the distracting or motivating potential of monetary incentive: “I found the possibility of (*Control*: getting feedback; *Loss*: losing money) to be distracting;” “I found the possibility of (*Control*: getting feedback; *Loss*: losing money) to be motivating” (see Supplementary Material S4 for full questionnaire).

#### Intrinsic Motivation Inventory

The Intrinsic Motivation Inventory (IMI) is a standard 22-item questionnaire assessing participants’ subjective experience regarding a task in an experiment (Ryan, 1982). After completing the task and SAMQ, participants completed the IMI, indicating how true each statement was for them during the LNS task (1 = *not all true*; 7 = *very true of me*). This inventory has four subscales: Interest/Enjoyment, Perceived Choice, Perceived Competence, and Pressure/Tension. Due to a misunderstanding regarding different versions of the IMI, the additional “Effort” scale also used by Hess et al. (2016) was unfortunately omitted. Interest/Enjoyment is often used as a self-report measure of intrinsic motivation.

### Procedure

Participants completed informed-consent procedures, a health and demographic survey, and the PAC questionnaire. Participants then received instructions for the LNS task and completed a practice run consisting of five trials of set sizes of 2–5. Participants had to get more than 80% correct on the practice trials to proceed to the main task. If not, they repeated the practice. Failure to reach criterion within three practice runs terminated the session (*n* = 5 older adults).

After the practice run, participants in the loss condition were endowed with $24. This money was put on the table in front of them. They were told that it was theirs to keep for good performance (in addition to the hourly compensation for study participation), but that 50 cents would be deducted for every incorrect trial. Both performance feedback (percent incorrect) and incentive feedback (the amount of money lost) were given after each run. After that, the experimenter immediately removed the amount lost and placed the new amount on the table. Control participants were given performance feedback only. Participants next completed the NASA-TLX with reference to the run they had just completed.

After the final LNS run and corresponding NASA-TLX questionnaire, participants completed the SAMQ and IMI to assess their evaluation of their attention, motivation, and performance during the task as a whole. They next completed the MMSE (Cockrell and Folstein, 2002; older adults only) and AD8 (Galvin et al., 2005; older adults only), and Extended Range Vocabulary Test (ERVT; Ekstrom, 1976), and were thanked, debriefed, and given the hourly compensation for their participation.

### Analyses

Analyses were conducted using R version 3.6.1 (R Core Team, 2017). Our overall analysis strategy followed that of Hess et al. (2016) in examining effects of age group and set size, with the additional between-subjects variable of incentive condition (control, loss). As described below, we also used correlation analyses to assess the relative accuracy of participants’ metacognitive reports.

See Table 2 for an overview of the predictions from each of the theoretical perspectives described in the Introduction; critical hypotheses are discussed in more detail below. The primary questions were whether the loss incentive would affect the dependent measures of performance, motivation, and metacognition, and whether incentive effects on these variables would interact with age and/or set size. A secondary question was whether we would replicate the age group and set size effects reported by Hess et al. (2016), especially for participants in the control condition (see Supplementary Material for these analyses). In some cases, especially for unexpected findings, we conducted additional *post hoc* analyses to provide potentially converging or disconfirming evidence or to give insight into potential mechanisms.

**TABLE 2:** An overview of the predictions from each of the theoretical perspectives.

**Perspective**	**Actual performance**	**NASA-TLX measures**	**SAMQ and IMI**	**Other**
**“Intuitive” view** (greater motivation and cognitive control under incentive)	Better in incentive condition	**Performance:** More accurate metacognition in incentive condition **Demand:** Lower in incentive condition **Effort:** Higher in incentive condition **Frustration:** No strong predictions; loss may lead to greater frustration at higher set sizes	Greater motivation in incentive condition Weak prediction for greater pressure/tension in incentive condition	

**Motivational shift** (older adults especially motivated by losses)	Generally the same as the “intuitive” hypothesis but with larger effects for older adults			

**Heuristic positivity effect** (older adults ignore negative information including losses)	Generally, the opposite of the “motivational shift” hypothesis; older adults *less* responsive to the loss incentive. Potentially less accurate metacognition (NASA-TLX Performance and IMI Perceived Competence) for older adults in the loss condition, if they are ignoring loss-related feedback.			

**Nuanced positivity effect** (older adults have reduced proactive, increased reactive responses to negative information; potentially followed by reframing)	Reduced performance for older adults in loss condition	**Demand:** Higher in loss condition **Effort:** No differences or reduced for older adults in loss condition **Frustration:** Increased by loss	Reduced motivation for older adults in the loss condition Reframing may inflate IMI Competence scores	Reframing may reduce long-term metacognitive accuracy for older adults in the loss condition

**Incentive as cognitive load**	Reduced performance under loss incentive, especially for older adults and at higher set sizes	**Performance:** If performance monitoring competes with the task itself for cognitive processing, ratings may be less accurate under loss incentive, especially at higher set sizes. **Demand:** Higher in loss condition, especially for older adults and at higher set sizes	Increased self-reported distraction in loss condition	

**NASA-TLX, NASA Task Load Index; SAMQ, State Attention and Motivation Questionnaire; IMI, Intrinsic Motivation Inventory.**

#### LNS Task Performance and Subjective Task Load (NASA-TLX)

The LNS data were analyzed using a mixed ANOVA design, with incentive and age group as the between-subjects variables and set size as the within-subjects variable. Greenhouse–Geisser corrected *df*, *F*, and *p-*values are reported where the sphericity assumption was violated. For easier reading, *df* values are rounded to the nearest integer in the text.

As in Hess et al. (2016), the NASA-TLX data were analyzed using multilevel modeling (MLM), rather than ANOVA, because the questions were consistently presented in the same sequential order, making the scales non-independent^1^. Included predictors were age group (young adults = referent), incentive condition (control = referent), linear and quadratic trends of set sizes (centered at 5.5), and all interaction terms. To control for individual variability, we included the random intercept for each individual (Field et al., 2012).

#### Posttask Motivation

The SAMQ questions regarding distraction (Q5) and motivation (Q6) were of primary interest for the present study; the other questions were included to be consistent with other publications from our lab that have used the questionnaire (Berry et al., 2014a, b; Lin et al., 2019), allowing interested readers or eventual meta-analyses to compare across experiments and study populations. The IMI subscales were used as posttask, holistic measures of participants’ metacognition and emotional–motivational response to the task, as compared to the run-specific questions presented by the NASA-TLX. Both the SAMQ and IMI subscales were analyzed using ANOVA with incentive condition and age group as between-subjects variables.

#### Correlations Between Questionnaires and Task Performance

The NASA-TLX “Performance” scale asked participants to rate their performance on a 0–100 scale immediately after completing the run and receiving feedback. It therefore provides a relatively specific, “in the moment” assessment of the participants’ metacognitive judgment of their performance. The IMI “Competence” scale measures a similar construct, but posttask, and in a more general sense (sample questions: “I think I did pretty well at this task, compared to my peers;” “I am satisfied with my performance on this task”). We used correlation analyses to examine whether age or incentive changed the relationship between these measures (NASA-TLX Performance and IMI Competence) and actual performance. Correlations between these measures and actual performance provided an estimate of participant’s *relative* metacognitive accuracy. That is, stronger positive correlations between these measures and actual performance would indicate that those individuals who gave themselves high ratings relative to others in their group did in fact tend to obtain higher scores than others in their group. Fisher’s *z* tests were used to test our *a priori* question of potential differences in correlation strengths between the groups.

The NASA-TLX Performance scale, with a range from 0 to 100, also allows for the calculation of *absolute* metacognitive accuracy, or the distance between a person’s actual performance, and their rating of their performance on the NASA-TLX scale (e.g., if four people all had an actual score of 75% correct, those rating themselves at either 77 or 73 would have better absolute accuracy than those rating themselves at 65 or 85). To measure this, we calculated a “metacognitive difference score” for each run by subtracting the participant’s NASA-TLX Performance rating on that run from their actual performance. The metacognitive difference scores were analyzed using the same MLM design as used to analyze the NASA-TLX scales. We included this as a *post hoc* analysis to explore the unexpected finding that participants in the loss condition gave themselves higher ratings for performance. However, in hindsight, it provides an additional test of the version of the “positivity effect” sometimes used to explain the results of previous studies: If older adults in the loss condition are ignoring the feedback information provided at the end of each run, they should be less accurate than the other groups.

## Results

### Loss Incentives Increase Perceived Performance but Not Actual Performance in the Working Memory Task

**FIGURE 1:**
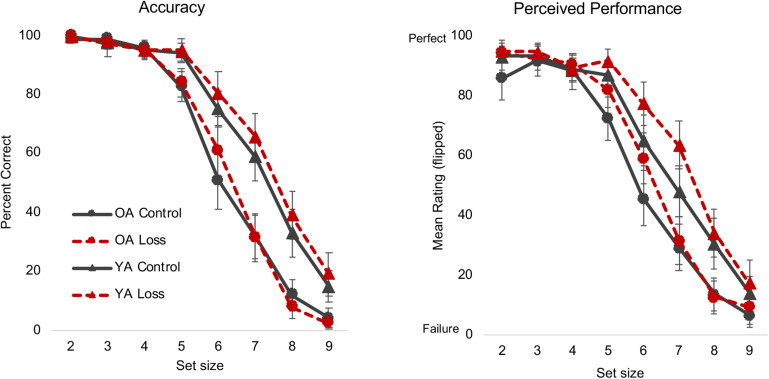
Letter Number Sequencing (LNS) accuracy and NASA-TLX perceived performance ratings. Different colors/lines (control = black solid line, loss = red dashed line) and shapes [triangle = young adults (YA), circle = older adults (OA)] are used to highlight the different conditions. Error bars represent 95% confidence intervals. The loss incentive did not affect actual performance **(left panel)** but did increase participants’ self-report ratings of their perceived performance **(right panel)**. See text and Table 3 for statistical details for this and other figures. NASA-TLX, NASA Task Load Index.

Loss incentive did not affect LNS performance, *F*(1, 159) = 1.27, *p* = 0.262, $\eta_{\text{p}}^{2}$ = 0.008, nor did it interact with age, *F*(1, 159) = 0.56, *p* = 0.455, $\eta_{\text{p}}^{2}$ = 0.003, or set size, *F*(4, 159) = 1.26, *p* = 0.281, $\eta_{\text{p}}^{2}$ = 0.008 (Figure 1). We replicated commonly observed set size and age effects and interactions: Accuracy decreased as set size increased, *F*(4, 159) = 879.29, *p* < 0.001, $\eta_{\text{p}}^{2}$ = 0.84; older adults showed lower accuracy compared to young adults, *F*(1, 159) = 67.80, *p* < 0.001, $\eta_{\text{p}}^{2}$ = 0.29; and older adults’ accuracy decreased at earlier set sizes than young adults’, *F*(4, 159) = 26.88, *p* < 0.001, $\eta_{\text{p}}^{2}$ = 0.14.

As an exploratory analysis of potential incentive effects on metacognition, we examined participants’ self-ratings on the Performance subscale of the NASA-TLX, administered after each run. The full MLM results for the Performance subscale and all NASA measures can be found in Table 3. To briefly summarize the critical results, in contrast to the lack of incentive effects on actual performance, participants in the loss condition perceived themselves to be more successful in accomplishing the task than did those in the control condition, β = 8.28, *t*(165) = 2.66, *p* < 0.01 (Figure 1).

**TABLE 3:** NASA-TLX MLM results (β).

**Effect**	**Mental demand**	**Physical demand**	**Temporal demand**	**Performance**	**Effort**	**Frustration**
Intercept	45.69***	9.32***	32.36***	74.20***	44.67***	24.97***
Age	−0.49	5.95*	7.26	−14.12***	2.56	9.06*
SS_linear_	10.08***	1.00***	7.30***	−12.07***	9.46***	5.40***
SS_quadratic_	0.33	0.09	0.77***	−1.76***	0.34	0.35
Age × SS_linear_	0.51	1.59***	2.77***	−1.64**	1.04*	3.93***
Age × SS_quadratic_	0.32	0.08	−0.12	0.63*	0.47	0.02
Incentive	−2.49	−2.71	−1.09	8.28**	0.65	3.19
Age × Incentive	−0.37	−2.70	−0.66	−1.86	−4.24	−1.95
SS_linear_ × Incentive	1.11*	0.55	−0.86	0.90	0.65	1.53**
SS_quadratic_ × Incentive	0.24	0.20	−0.01	−0.54	0.03	−0.04
Age × SS_linear_ × Incentive	−0.28	−1.59**	0.36	−1.43	−0.60	−0.92
Age × SS_quadratic_ × Incentive	0.22	−0.09	0.25	0.24	0.05	0.31

***p < 0.001, **p < 0.01, *p < 0.05.*NASA-TLX, NASA Task Load Index; MLM, multilevel model; SS, set size.*

The results so far indicate that loss incentives do not improve performance, contradicting the intuitive hypothesis. As we describe in *Discussion*, in hindsight, this may not be surprising given the task constraints (relatively fast presentation of stimuli, verbal response required on every trial) and that several other studies have failed to find incentive effects on performance; Hess et al. (2016) also did not find effects of an alternative motivation manipulation on this same task. More importantly, we did not find any evidence in either actual or perceived performance that older adults were any more (motivational shift hypothesis) or less (heuristic positivity effect hypothesis) sensitive to the loss incentive.

The higher Performance self-ratings in the loss condition were an unexpected finding, which we discuss in the context of the other metacognitive measures below. Before turning to those issues, we review the results for the other NASA-TLX subscales and posttask questionnaires.

### Loss Incentives Increase the Perceived Demands and Frustration at Higher Task Loads but Not the Effort to Meet That Demand

The main measures of interest for the NASA-TLX were the Mental Demand, Effort, and Frustration subscales. Hess et al. (2016) noted that the Mental Demand and Effort scales were especially related to the construct of engagement, both in terms of face validity and in their ability to predict a physiological measure of engagement [systolic blood pressure (SBP) reactivity]. As noted in Table 2, an intuitive “incentive increases motivation” perspective predicts that incentive should increase the effort people put in to maintain performance as actual demand (set size) increases and may also reduce perceived demand (i.e., people may perceive the task as less demanding if they are strongly motivated). In contrast, a “disengagement” perspective predicts a lack of willingness to increase effort in response to an increase in perceived demand (The “positivity effect” view does not make obvious predictions for these measures).

**FIGURE 2:**
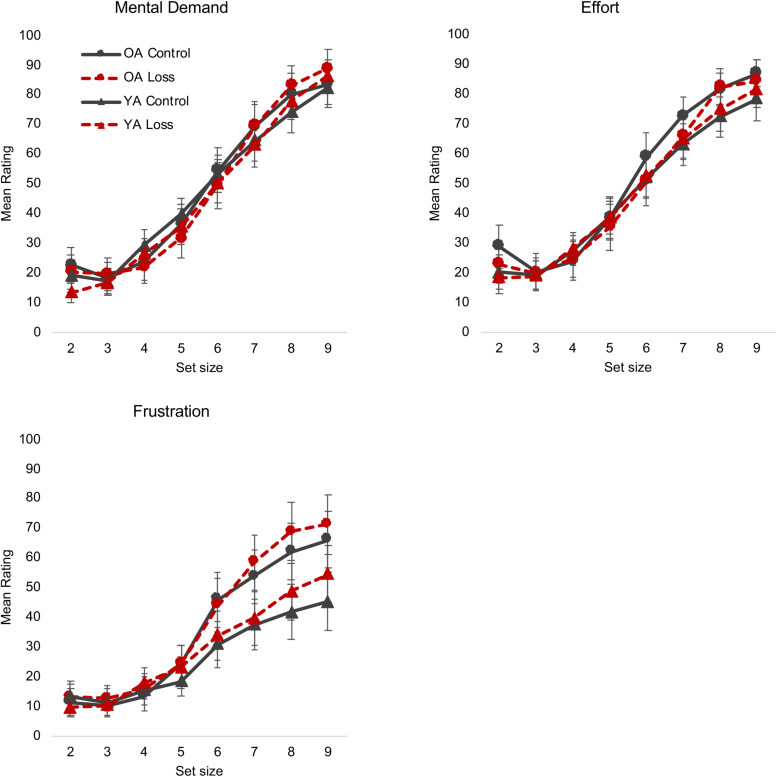
NASA-TLX mental demand, and effort, and frustration. Different colors/lines (control = black solid line, loss = red dashed line) and shapes [triangle = young adults (YA), circle = older adults (OA)] are used to highlight the different conditions. Error bars represent 95% confidence intervals. The loss incentive increased participants’ reports of mental demand and frustration but did not increase effort to meet those demands. NASA-TLX, NASA Task Load Index.

The results were more consistent with the disengagement perspective. For the Mental Demand measure, the incentive × set size interaction was significant (Table 3) with participants in the loss condition giving numerically lower ratings of demand until about set size 6 and giving numerically higher ratings from set size 8 (Figure 2; see also Supplementary Material S2, which shows the results more clearly by collapsing across age group). *Post hoc t* tests suggested that this interaction is due to a significant increase in ratings from set size 8 to set size 9 in the loss group [*t*(168) = −2.35, *p* = 0.019], but not in the control group [*t*(166) = −1.71, *p* = 0.087). In contrast, for the Effort measure, there was no effect of incentive (Table 3). In other words, despite perceiving greater demand, participants in the loss condition were not inclined to increase effort to meet that demand.

We were also interested in the Frustration subscale, as the “positivity effect” view would make different predictions than the other two perspectives. That is, if older adults ignore or downplay negative information in the service of regulating emotion, they might be expected to show less frustration than young adults (especially in the loss condition) at the higher set sizes, when errors and thus losses are more likely. The “disengagement” perspective predicts a different chain of events: The feedback and loss information immediately after the trial is relatively difficult to ignore or avoid, and a resulting increase in frustration would be predicted to lead to subsequent, downstream disengagement. The “incentive increases motivation” viewpoint might also predict increased frustration, if that motivation or desire to achieve/retain reward is literally frustrated by the increase in errors, and thus losses, at higher set sizes (Carver and Harmon-Jones, 2009; Angus and Harmon-Jones, 2019).

For the Frustration subscale, set size had significant interactions with both incentive and age group. The three-way interaction was not significant (Table 3). In both cases, the two groups (young vs. old; loss vs. control) were largely identical at the lower, easier, set sizes, with larger differences between the groups appearing at the higher, more difficult set sizes (Figure 2). Age group differences in particular closely paralleled the accuracy data in when they began to show a separation (i.e., older adults had low Frustration scores for set sizes 2–4 and began to show an increase around set size 5, whereas for young adults, the sharper increase occurred around set size 6). In short, these data support the idea that the loss incentive increases frustration specifically at higher set sizes when errors are more likely to occur, and there is no evidence that older adults are either immune to or especially sensitive to this effect.

The other subscales were not as incisive theoretically but are reported (Table 3 and Supplementary Materials S1, S3) for completeness, including comparison with the prior study by Hess et al. (2016).

### Loss Incentives Increase Distraction in Young Adults and Decrease Motivation in Older Adults

**FIGURE 3:**
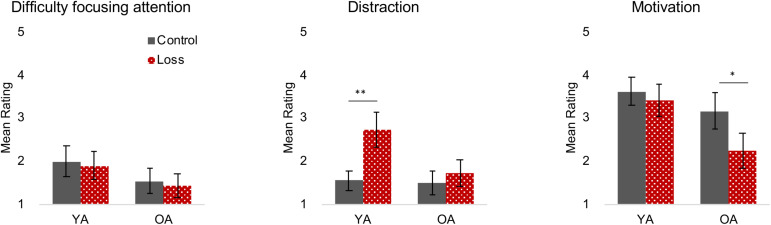
State Attention and Motivation Questionnaire (SAMQ) (Q4–Q6). Different colors/patterns (control = black filled, loss = red dotted) are used to highlight the different conditions for young (YA) and older adults (OA). Error bars represent 95% confidence intervals. **p < 0.001, *p < 0.01. Loss incentive increased distraction for young adults and decreased motivation for older adults.

Figure 3 shows the results of directly asking participants about their focus of attention and the degree to which the feedback or incentive was distracting or motivating. Older adults gave lower ratings for difficulty focusing attention than did young adults, replicating counterintuitive but typical findings in the literature, *F*(1, 160) = 8.47, *p* = 0.004, $\eta_{\text{p}}^{2}$ = 0.05.

A significant age × incentive interaction for the distraction question indicated that young and older adults had different reactions to the loss incentive feedback, *F*(1, 160) = 8.51, *p* = 0.004, $\eta_{\text{p}}^{2}$ = 0.049. Young adults under loss incentive reported higher distraction than those in the control condition, *t*(83) = −4.89, *p* < 0.001, but this effect was not observed in older adults, *t*(82) = −1.08, *p* = 0.285. For the motivation question, we observed a significant incentive effect, *F*(1, 160) = 8.25, *p* = 0.005,$\,\eta_{\text{p}}^{2}$ = 0.05 where those under loss incentive show lower motivation. Although the age × incentive interaction was not significant, *F*(1, 160) = 3.40, *p* = 0.067,$\,\eta_{\text{p}}^{2}$ = 0.02, the incentive effect was largely driven by older adults, *t*(82) = 3.08, *p* = 0.003, and not significant for young adults, *t*(83) = 0.80, *p* = 0.428.

One caveat to these results is that they reflect participant’s answers to the direct questions about their responses to the incentive and feedback. We did not see incentive effects on the more general measures provided by the IMI, including the Interest/Enjoyment scale (Supplementary Material S5). This may be due to the less targeted nature of the IMI questions and their focus on how fun, interesting, or enjoyable the task is rather than the participant’s inner motivation or desire to do well.

### Loss Incentives Improve the Accuracy of Immediate, Absolute Metacognitive Judgments, but May Distort Relative Judgments of Competence for Older Adults

**FIGURE 4:**
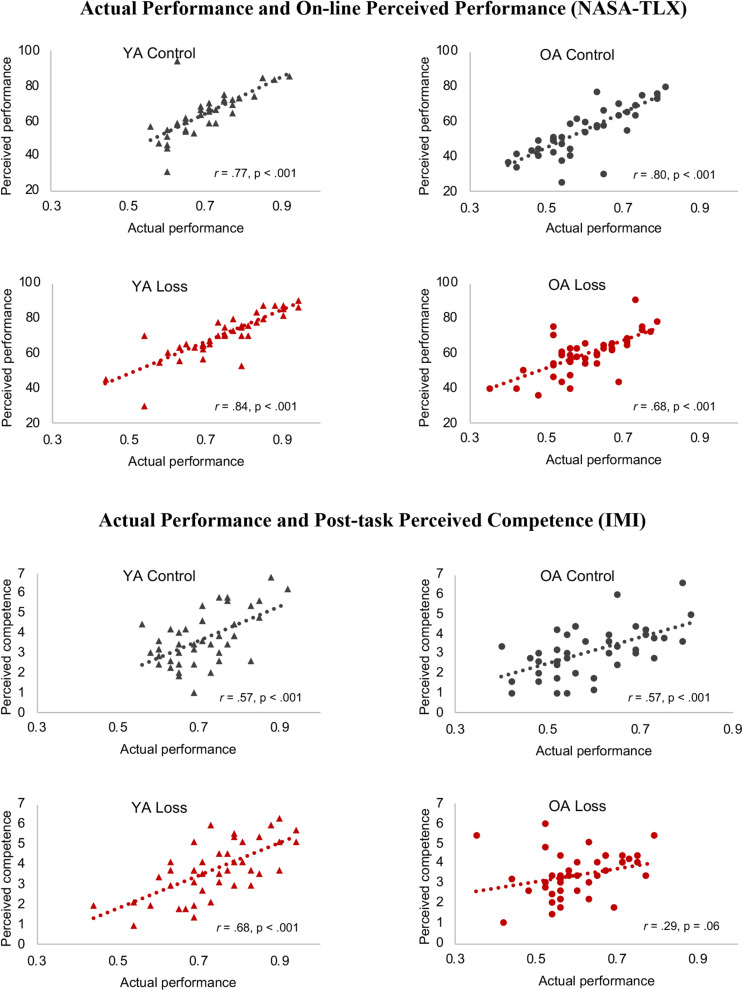
*Relative* metacognitive accuracy. Different colors (control = black, red = loss) and shapes [triangle = young adults (YA), circle = older adults (OA)] are used to highlight the different conditions. Correlations between actual and perceived performance were moderately strong for all four groups. A different pattern emerged for correlations between actual performance and IMI Competence ratings: this correlation was only marginal for older adults in the loss condition, while the other groups maintained moderate correlations. NASA-TLX, NASA Task Load Index; IMI, Intrinsic Motivation Inventory.

We next conducted further exploratory analyses of how the loss incentive might affect participants’ metacognitive judgments. The hypothesis that older adults ignore negative information predicts that older adults in the loss condition would have a weaker relationship between their actual and perceived (self-rated) performance. This was not the case for the Performance subscale of the NASA-TLX: Correlations between perceived and actual performance were moderately strong for all four groups (all *r* = 0.68, *p* < 0.001; Figure 4, top panel).

**FIGURE 5:**
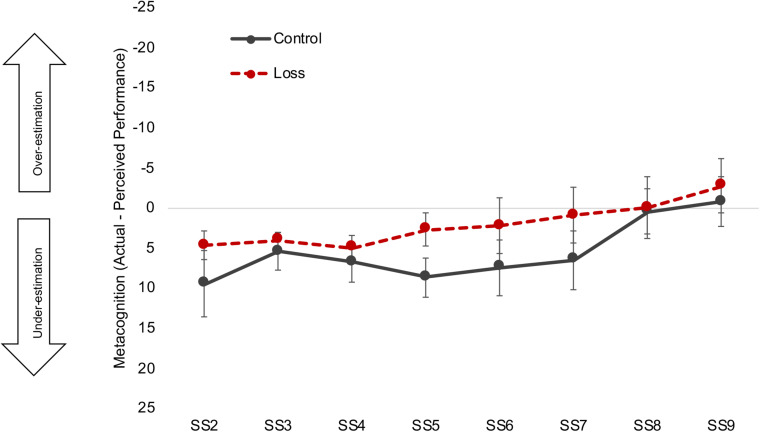
*Absolute* metacognitive accuracy. Black solid line and red dashed line denote control and loss condition, respectively. Error bars represent 95% confidence intervals. Participants in the loss incentive condition had less discrepancy between their perceived and actual performance than did participants in the control condition, with group differences largest at the intermediate set sizes (see text and Supplementary Table S6 for statistical details). Note: Scale on *y*-axis is reversed for ease of interpretation. Zero means accurate judgment. See Supplementary Material S7 for the full age × incentive graph. SS, set size.

Moreover, the metacognitive difference scores (actual performance - self-rated performance) were analyzed using the same MLM design as used to analyze the NASA-TLX scales (see Supplementary Material S6 for the full results). The results showed that both younger and older adults in the loss condition in fact showed less discrepancy between their actual performance and perceived performance than did their counterparts in the control condition, β = −4.84, *t*(165) = −2.43, *p* = 0.016 (Figure 5). There was also a significant quadratic interaction between set size and incentive condition, β = 0.45, *t*(1175) = 2.22, *p* = 0.026. Both the control and loss groups tended to underestimate their performance in the lower set sizes and get close to accurate judgment or slight overestimation at the higher set sizes. The discrepancies between the groups appear to be greatest at the middle set sizes (4–7), where the loss incentive group’s ratings underestimated their performance less than did those of the control group. Full MLM results for metacognitive difference scores are shown in Supplementary Material S6.

A different pattern emerged for the IMI Competence rating, which was given after the entire task (rather than immediately after run feedback) and focused on participants’ overall satisfaction with their performance and whether they felt they had performed well in comparison with their peers. While the other three groups maintained moderate correlations between this measure and their actual performance, this correlation was only marginal for older adults in the loss condition, *r* = 0.29, *p* = 0.061 (Figure 4). This was significantly smaller than the correlation between their NASA-TLX Performance rating and actual performance (modified Fisher’s *z* test, *z* = 2.37, *p* = 0.009; Steiger, 1980; calculation tool provided by Lee and Preacher, 2013). For the other groups, the correlations between IMI Competence and actual performance remained in the moderate range, all *r* ≥ 0.57, *p* < 0.001. Comparing across groups, Fisher’s *z* tests showed that the correlation for older adults in the loss condition was significantly weaker than that of the young adults in the loss condition (*p* = 0.009), marginally so compared to the other two groups (both *p* = 0.06).

## Discussion

We examined the effects of a loss-based incentive on young and older adults’ working memory performance, motivation, and metacognition. Incentive did not impact performance, but instead increased participants’ perceptions of mental demand and their frustration at the higher, more demanding set sizes. The loss incentive also increased the absolute accuracy of immediate metacognitive judgments, that is, participants’ ratings of how well they did compared to their actual performance. These results are not consistent either with the “incentive increases motivation” or the heuristic “older adults ignore loss information” hypotheses. Older adults were at least as sensitive to loss information in the immediate postrun ratings as were young adults, and their immediate postrun metacognitive performance ratings were particularly accurate in the loss condition, suggesting close attention to the loss incentive feedback.

The results did not completely fit any of the predictions outlined in Table 2, but overall seemed most consistent with the idea that, especially at the highest set sizes when errors were most common, loss incentive increased the perceived “costs” (mental demand, frustration) of performance. Somewhat contrary to the suggestion that older adults may be more sensitive to unavoidable negative information and/or more sensitive to such costs (c.f., Charles, 2010; Hess, 2014), the effects appeared to be of similar size for younger and older adults. However, other aspects of the results suggest that these equivalent effects occurred for different reasons, with the loss incentive being more distracting to young adults, more demotivating to older adults. The change in metacognitive accuracy by older adults in the loss condition from immediate, specific performance judgments vs. later judgments of competency in the task as a whole also seems consistent with the suggestion that, when negative information is unavoidable in the moment, older adults may instead cope by reframing later on (Charles, 2010).

Despite their increased perception of demand and frustration, as well as more accurate judgments of performance, participants in the loss condition did not increase their effort to meet that demand and improve their performance. To further explore the possibility that, for older adults, this failure to increase effort might be related to disengagement and decreased motivation, we conducted additional exploratory analyses examining correlations between changes on the NASA-TLX Effort scale from the lowest (2) to highest (9) set size and the posttask question about motivation [*p*-values corrected for multiple comparisons using the false discovery rate (FDR) approach because of the exploratory nature of the analyses]. The relationship between effort and motivation change went in the opposite direction for older adults in the control and loss conditions, Fisher’s *z* = 2.12, *p* = 0.034. However, this result should be considered only suggestive and interpreted with caution given the exploratory nature of the analyses and that the individual correlations did not reach significance (control condition Kendall rank correlation coefficient *tau* = 0.22, *p*_FDR_ = 0.14; loss condition *tau* = −0.25, *p*_FDR_ = 0.14). The loss-reversal pattern appears to be specific to older adults and to the motivation measure: Correlations for young adults did not approach significance (all *p*_FDR_ > 0.40), and in the older adults, the control and loss incentive groups showed similar correlations between distraction ratings and increases in effort (control *tau* = −0.38, *p*_FDR_ = 0.006; loss *tau* = −0.26, *p*_FDR_ = 0.034).

In addition, although it had not been part of our thought process in setting up the correlation matrix, we also observed that for the control groups, motivation and distraction tended to be negatively correlated (*tau* = −0.35, *p*_FDR_ = 0.021 for young adults; *tau* = −0.25, *p*_FDR_ = 0.07 for older adults) with the opposite pattern in the loss groups (*tau* = 0.23, *p*_FDR_ = 0.07 for young adults; *tau* = 0.55, *p*_FDR_ < 0.001 for older adults). This again seems inconsistent with the idea that older adults ignored the negative loss incentive information. Instead, for both age groups, the more motivated they were by the loss incentive information, the more distracting they found it.

### Performance vs. Subjective Measures

Contrary to initial expectations, we did not see either beneficial or detrimental effects on performance by either group. Figure 1 suggests a very small numerical advantage for the loss condition, but even at the set size with the largest difference, the effect is quite small (*d* = 0.24) and most likely noise. We originally chose this task because Hess et al. (2016) had found age and set size differences in a physiological measure of engagement during the task. An earlier set of studies in our lab found that loss incentive reduced older adults’ performance on a measure of focused attention and increased their self-reported mind wandering (Lin, 2018; Lin et al., 2019), and so we had thought we might see similar effects here.

Of course, it is possible that our loss incentive manipulation was simply ineffective and inadequate. A reviewer raised the question of whether this might be the case because of the between-subjects design and whether a within-session contrast with reward or neutral trials might be necessary to make the loss salient produce an effect. Although that explanation cannot be ruled out, we think it is unlikely to be the case. First, there are the findings of effects on the subjective measures, suggesting that the loss incentive was indeed salient and that the lack of effects on working memory performance were due to a lack of sensitivity in the measure. Other studies suggest that between-subjects incentive manipulations can affect performance in older adults: Barber and Mather found crossover interactions for between-subjects manipulations of stereotype threat and gain/loss incentive on both working memory and clinical cognitive assessments (Barber and Mather, 2013; Barber et al., 2015). As we have already noted, other datasets from our lab show that older adults’ performance can be impaired by similar between-subjects incentive manipulations, although these findings should be considered preliminary until they have undergone full peer review and publication (Lin, 2018; Lin et al., 2019; see also Jang et al., 2020).

Instead, although targeted experiments will be required to test it, our working hypothesis is that discrepancies across studies, whether they show performance differences as a result of incentive, especially loss incentive, may be heavily influenced by differences in the task constraints and proactive control requirements. Incentives appear to largely affect the engagement of proactive control (Chiew and Braver, 2016; Mäki-Marttunen et al., 2019; general reductions in response time may be an exception). The focused-attention task used in our earlier study made strong demands on self-initiated, proactive processing (rare targets and responses, low-salience targets distinguishable only by their duration). The LNS task uses a relatively fast presentation of to-be-remembered stimuli (one per second) and requires a verbal response on each trial – literally requiring the participant to “engage with” the experimenter. Thus, it may rely more on reactive control; the low ratings of mind wandering and difficulty focusing attention seem consistent with that interpretation. Future experiments that specifically isolate task constraints and top–down control requirements will be needed to determine the plausibility of this interpretation.

On the other hand, the lack of performance differences helps to alleviate concerns that the effects we see on the subjective measures are simply downstream artifacts of poor performance. That is, it is difficult to say that the higher mental demand ratings (for example) by participants in the loss condition are simply an attempt to “excuse” lower performance, since they did not in fact have lower performance.

We also examined whether the end-of-task measures might be especially influenced by the last few runs. This was the case for the IMI competence measure, as might be expected, given that the final runs are also the ones where performance is most difficult and competence becomes a question: For all groups except the older adult loss group, correlations between performance and the IMI Competence ratings were higher for the last three set sizes (*r* = 0.36–0.60) than for the first three set sizes (*r* = −0.31–0.31). For the older adult loss group, correlations were consistently low (*r* = −0.06–0.17 for the first three set sizes; *r* = 0.07–0.27 for the last set sizes), as would be expected from the results shown in Figure 4. There were no systematic changes in correlation with set size for the SAMQ Motivation or Distraction questions, or IMI Interest/Enjoyment measures, especially for the incentive groups. [The young adult control group showed hints of such a pattern for the IMI Interest/Enjoyment measure (*r* = −0.06–0.28 for the first three set sizes; *r* = 0.13–0.36 for the last three), but given fluctuations across the set sizes, this seems unlikely to be meaningful.] Thus, there is no evidence that the end-of-task measures of motivation and distraction were unduly influenced by the last few runs/highest set sizes.

The opposite critique may come to mind when considering age differences: Young adults had better performance than older adults. Of course, that is also the case in most previous studies of age × incentive interactions in cognitive control tasks. The present task has the advantage that the range of set sizes used here allows us to examine the issue, at least for the postrun NASA-TLX ratings. We did a follow-up analysis using only those set sizes where performance for young and older adults was equivalent (between 25 and 75% accuracy; set sizes 5–7 for older adults; set sizes 6–8 for young adults; rescaled as “low, medium and high” for each group). In that case, the Mental Demand and Effort ratings were generally higher for young adults, whereas Frustration remained somewhat higher for older adults. It did not introduce any new age × incentive interactions compared to the analyses reported above, although there was a trend for the Effort ratings of older adults in the loss condition to be especially low. In general, comparing the restricted-range results to the full dataset suggests that incentive effects overall were greatest at the highest set sizes, when load exceeded capacity, but there was no suggestion of interactions with age or that age differences in performance played a role.

### Limitations and Comparisons (or the Lack Thereof) With Previous Studies

There are several limitations and differences from other studies that should be kept in mind when interpreting these results and their place in the literature, as well as strengths and weaknesses that are shared with other studies in this field. First, we focused on loss incentives because they are understudied; losses are thought to be increasingly important in later life (Baltes et al., 1999), the opportunity to avoid losses is often used to motivate older adults, and this is the condition that is most theoretically incisive: The general/intuitive “incentive increases motivation and thus attention and performance,” heuristic positivity effect (“older adults ignore negative information”), and nuanced positivity effect/disengagement hypothesis all make similar predictions for reward conditions. The “incentive as cognitive load” makes similar predictions for reward and loss incentive. Prior studies that did examine both reward and loss effects on cognitive performance in young and older adults have already found patterns contradicting the “motivational shift” hypothesis, which appears to apply to more general orientations and choice behaviors, and possibly to avoidance learning.

It is the case that we cannot rule out that “gain” incentives would have had similar results in the present study; the complementary criticism applies to the majority of studies that have focused solely on gain incentives. Behavioral (O’Brien and Hess, 2019) and neural (e.g., Paschke et al., 2015; Cubillo et al., 2019) evidence suggests that gain and loss operate through partially independent processes. However, this issue needs further examination, and in general, studies in this field would benefit from including both conditions. What we can say is that we did not find any evidence that loss incentive generally improved performance and motivation and that older adults appeared to be at least as responsive to the loss incentive as were young adults.

Second, as stated earlier, it was explicitly *not* our intention to do another incremental variation on existing studies that, besides focusing on gain effects, have with rare exception used trial-wise manipulations on cognitive control tasks. We instead wanted to take the first step in addressing several important but understudied questions, not only of incentive type (loss, as noted above) but also of cognitive domain (working memory) and session-wide implementation of incentives. While the differences in our approach make it difficult to compare our results directly to existing laboratory studies, we believe that this last aspect is especially important, given how performance incentives are typically implemented in everyday life. Trial-wise implementations have an advantage in statistical power, but this may come at the cost of generalization to real-world situations (c.f., Deci et al., 1999; Cerasoli et al., 2014).

Another reason we have specifically avoided trial-by-trial incentives in our studies is that the changing incentive cues and delivery of reward/loss information on every or almost every trial are likely to drive attention and engagement in the “bottom–up” fashion described earlier. Several studies have already found different incentive effects for block- or run-wise implementation of incentives vs. trial-wise manipulations (Jimura et al., 2010; Paschke et al., 2015; Bruening et al., 2018); differences from session-wide effects may be even more pronounced (Lin, 2018). Although they examined downstream effects of correct/error and gain/loss feedback on incidental encoding during a previous task rather than incentivized performance, analysis by Mather and Schoeke (2011) suggest that trial-history effects could be an interesting compromise method to test whether, e.g., disengagement (or overarousal) builds up over multiple errors or losses (see also Schmitt et al., 2017). Regardless, it seems important to have both types of studies in the literature to see where effects converge or diverge and, in the latter case, to ultimately conduct targeted, parametric manipulations to understand why. We hope that the present findings will – to coin a phrase – provide some incentive to do so.

Third, our use of subjective response measures, especially examination of potential effects on metacognition, is relatively novel and provides further insights into the pathways by which incentives may have their effects. However, such measures come with their own limitations, including potential response bias, impression management, and so on. As noted above, although the lack of incentive effects on performance can be seen as a limitation in some respects, raising questions about whether the incentive manipulation was effective, on the other hand, has the advantage of alleviating the concerns that the loss groups’ higher ratings of mental demand, frustration, and distraction (young adults) or reduced motivation (older adults) might be attempts to “blame” poor performance on those factors in retrospect. Besides their preserved actual performance, participants in the loss condition also gave themselves higher and more accurate immediate self-ratings of performance, especially at the higher set sizes. It seems hard to reconcile this greater confidence and accuracy with the idea that they were more likely to use increased mental demand, frustration, distraction, or loss of motivation to excuse performance declines. Again, what we have here is a complementary set of advantages and disadvantages compared to studies that have examined physiological or neural responses to incentive manipulations; what is ultimately needed is a combined approach.

Another critique that can be applied both to this study – and almost every other study of age × incentive effects, including many of the others in this Frontiers Research Topic – is “maybe older adults just don’t care (as much) about the money.” This seems a bit hard to reconcile with the equivalent effects of the incentive on young and older adults for many of our measures. However – although it should be considered exploratory – the different patterns shown by young and older adults for the posttask distraction vs. motivation questions suggests that there may be at least some truth to this. In a larger sense, we agree entirely that older adults, at least those who are likely to participate in studies in our lab and the labs of other university-based investigators, are unlikely to find the money *per se* of primary interest. We suspect that, instead, the loss incentive in particular has its power by drawing attention to errors. We are beginning studies to test this possibility more directly. Providing some indirect support, Dhingra et al. (2020) reported less behavioral and neural sensitivity to incentive magnitude (dollar vs. cent) in older vs. young adults. However, in the case of losses, this was due to a relatively higher response to even small losses in older adults. Another important question for this area of study more generally is how different incentive amounts and types may affect results, and potentially interact with participant demographics.

Finally, an aspect of the present study lacking in many others was our examination of subjective measures, both immediately and posttask. It is interesting that younger and older adults showed similar incentive effects for the ratings of mental demand, performance, and frustration taken during the task, with age differences emerging in the more holistic, posttask measures. This could be seen as consistent with claims that older adults may be just as affected as young adults by unavoidable negative information “in the moment,” but more likely to respond to it with more passive strategies, and by later reframing or reappraising the situation to put it in a more positive light (e.g., Charles, 2010). Future studies using instruments designed to more systematically explore how metacognition and the emotional/motivational response to incentives is affected by the specificity (atomistic vs. holistic) and temporal (during/immediately after performance vs. somewhat later on) dimensions, as well as their interaction, will be important for more definitively identifying which factors exert a critical influence over these effects.

### What Are the Roles of “Engagement” and Task Constraints in Studies of Incentive?

As noted in *Introduction*, incentives are often used (or assumed) to increase proactive control in an effort to improve performance (Botvinick and Braver, 2015); the “engagement” idea of Hess and colleagues (Ennis et al., 2014; Hess, 2014) is similar. This leads to the question of how to define “engagement.” Although Hess’s theoretical writings have not specifically addressed issues of top–down (proactive, goal-related) vs. bottom–up (reactive, task or stimulus related) factors, he has noted that he means the term to be synonymous with “effort” and also emphasizes the idea of the choice whether or not to engage, which seems more consistent with the top–down interpretation. However, the degree to which engagement of this type is required likely varies inversely with the degree to which the task itself is inherently “engaging” because of constraints or stimuli that drive attention in a more bottom–up or reactive fashion. Several functional MRI (fMRI) studies indicate that incentives may have their primary effects on proactive, self-initiated control (e.g., whether participants engage frontoparietal regions at the point of a cue which would allow them to prepare for the upcoming probe, vs. waiting for the probe), although this has primarily been demonstrated for reward incentives (e.g., Jimura et al., 2010; Etzel et al., 2015; see Cubillo et al., 2019 for effects of loss incentives suggesting a shift to reactive control).

Putting this together with the boundary conditions on the positivity effect noted by Carstensen and colleagues, when loss information is unavoidable but task constraints are high, older adults may react to the negative information at a subjective and motivational–emotional level without this drop in motivational “engagement” decreasing performance. One interesting prediction is that higher task constraints should lead to preserved performance at the cost of greater subjective demand and frustration, whereas relatively unconstrained tasks provide an opportunity to reduce engagement and negative subjective experience but at the cost of reduced performance. This hypothesis regarding the potential role of task constraints should be regarded as that – a hypothesis – rather than a definitive conclusion.

An alternative, less process-specific explanation for the differences between the studies might be that the present task was simply more difficult, especially at the higher set sizes. However, this alternative runs into some complications given that, on the one hand, more difficult tasks typically decrease mind wandering (e.g., Baird et al., 2012; Konishi et al., 2015; see Seli et al., 2018 for discussion of exceptions) but, on the other hand, are usually considered to be exactly the situations in which incentive and motivation are likely to be most important (e.g., Botvinick and Braver, 2015; Ferdinand and Czernochowski, 2018; Kostandyan et al., 2019).

To our knowledge, there has not been a systematic investigation of how either incentive effects or the positivity effect may be impacted by changing the degree to which engagement is driven by “bottom–up” vs. “top–down” within the same task. One way to differentiate these ideas while controlling for task difficulty might be, e.g., comparing rare-response vs. frequent-response versions of the same attention task (c.f., Staub et al., 2015), or varying retention intervals in a working-memory task. This kind of task analysis and testing of parameters and boundary conditions may be an important direction for future research, especially as many real-world tasks are relatively unconstrained (e.g., reading, writing, participating in a conversation, driving) and thus may rely more on the top–down, self-initiated aspects of attention (Hess et al., 2011, 2018).

## Conclusion

The study of age differences in the response to incentives during cognitive challenging tasks is still at very early stages, although growing quickly. Thus far, most studies have used attention and cognitive control tasks, used reward incentives, and implemented incentive on a trial-wise basis. We took a complementary approach (working memory task, loss incentive, session-wide incentive implementation), with a complementary set of strengths and weaknesses in our methods, design, and the conclusions that can be drawn.

Our results suggest caution in generalizing the results of previous studies, especially to everyday life scenarios: They do not support the idea that incentive generally (i.e., regardless of valence) increases motivation and performance even for young adults, or that older adults ignore negative information provided by loss incentives. Another relatively novel aspect of our study was the inclusion of metacognitive and self-report measures of motivation, distraction, and related constructs. The loss incentive appeared to increase participants’ attention to their own performance, their perceptions of mental demand at higher set sizes, and their frustration at not being able to maintain good performance at those higher set sizes. Interestingly, these perceived increases in demand and frustration at higher set sizes were not met with concomitant increases in effort. Instead, young adults reported finding the incentive distracting, whereas older adults found it demotivating.

These results come with the usual caveats accompanying self-report measures, although supposedly more objective physiological measures have a complementary problem of somewhat subjective interpretation by the investigator (as opposed to the participant). That is, they are often related to some aspect of sympathetic arousal, but is this arousal indexing “engagement” or some other construct such as frustration or anxiety? Ideally future studies will combine these approaches; self-report measures may provide richer and more precise interpretations of the neural and physiological results, especially if combined with fine-grained analysis of performance results [e.g., response time, vigor (speed or force), or variability] and careful experiment construction to get at different cognitive, emotional, or motivational constructs. The role of individual and cultural differences in attitudes toward different types and levels of incentives is also an understudied topic. Finally, task constraints vs. the demand for proactive, self-initiated top–down control may be an important but as yet somewhat understudied factor in determining when and how incentives may affect performance and/or subjective responses.

In short, our study may raise as many questions as it answers. One of the most important questions it raises concerns the degree to which the results of previous studies can be generalized, especially to real-world scenarios. However, we believe that, in the long run, a careful consideration of issues related to proactive, top–down control vs. reactive, bottom–up attention will provide an important organizing principle for understanding the literature and driving it forward. We look forward to reading the other papers in this issue that will inform our own understanding of these issues, as well as future studies to test those ideas.

## Data Availability Statement

The datasets generated for this study will not be made publicly available as unfortunately we were not able to obtain permission from our IRB to share the data to a public repository. Data may be shared with other investigators upon request with a data use agreement and approval from the IRBs at both institutions.

## Ethics Statement

The studies involving human participants were reviewed and approved by the University of Michigan IRB. The patients/participants provided their written informed consent to participate in this study.

## Author Contributions

HJ and CL contributed to study design, data collection, data analyses, and manuscript writing. ZL contributed to study design. All authors contributed to the article and approved the submitted version.

## Conflict of Interest

The authors declare that the research was conducted in the absence of any commercial or financial relationships that could be construed as a potential conflict of interest.

## Footnotes

1 One might question whether the LNS runs were truly independent given previous findings suggesting that ascending set-size presentation leads to both practice effects, differentially affecting young adults, and proactive interference, differentially affecting older adults (e.g., May et al., 1999; Lustig et al., 2001; Rowe et al., 2008). As a precaution we also used MLM to analyze the LNS results; conclusions did not differ between the two methods.
