# Supplementary material for: Losing Money and Motivation: Effects of Loss Incentives on Motivation and Metacognition in Younger and Older Adults
Source: Front Psychol. 2020 Jul 15;11:1489. doi: 10.3389/fpsyg.2020.01489 (PMC7381126; doi:10.3389/fpsyg.2020.01489)
Supplement: Supplementary file 1 [file Data_Sheet_1.docx]

Supplementary Material for
“Losing Money and Motivation: Effects of Loss Incentives on
Motivation and Metacognition in Younger and Older Adults”

# Supplementary Figures and Tables

(From the following page)

**S1. NASA-TLX: All measures**


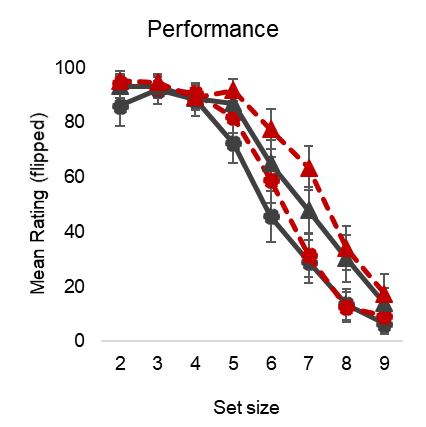


Failure

Perfect

Different colors/lines (control = black solid line, loss = red dashed line) and shapes (triangle = young adults (YA), circle = older adults (OA)) are used to highlight the different conditions. Error bars represent 95% confidence intervals. NASA-TLX: NASA Task Load Index

S2. NASA-TLX Mental Demand collapsed across age group

Different colors/lines (control = black solid line, loss = red dashed line) are used to highlight the different conditions. Error bars represent 95% confidence intervals. NASA-TLX: NASA Task Load Index

S3. NASA-TLX MLM results (*β*) in comparison with Hess et al. 2016

| Item | Mental demand | | | | Physical demand | | | | Temporal demand | | | | Performance | | | | Effort | | | | Frustration | | | |
| --- | --- | --- | --- | --- | --- | --- | --- | --- | --- | --- | --- | --- | --- | --- | --- | --- | --- | --- | --- | --- | --- | --- | --- | --- |
| Effect | Jang & Lustig | | Hess et al | | Jang & Lustig | | Hess et al | | Jang & Lustig | | Hess et al | | Jang & Lustig | | Hess et al | | Jang & Lustig | | Hess et al | | Jang & Lustig | | Hess et al | |
| Intercept | 45.69 | *** | 46.85 | *** | 9.32 | *** | 9.13 | *** | 32.36 | *** | 32.86 | *** | 74.20 | *** | 45.19 | *** | 44.67 | *** | 44.22 | *** | 24.97 | *** | 30.32 | *** |
| Age | -0.49 |  | 7.83 | * | 5.95 | * | 10.49 | ** | 7.26 |  | 8.91 | * | -14.12 | *** | 1.1 |  | 2.56 |  | 9.26 | * | 9.06 | * | 8.14 |  |
| SS_linear_ | 10.08 | *** | 11.85 | *** | 1.00 | *** | 2.24 | ** | 7.30 | *** | 7.81 | *** | -12.07 | *** | 11.86 | *** | 9.46 | *** | 9.7 | *** | 5.40 | *** | 8.35 | *** |
| SS_quadratic_ | 0.33 |  | -0.06 |  | 0.09 |  | 0.18 | * | 0.77 | *** | 0.19 |  | -1.76 | *** | -0.24 |  | 0.34 |  | 0.19 |  | 0.35 |  | 0.34 | * |
| Age × SS_linear_ | 0.51 |  | -2.34 | * | 1.59 | *** | 2.17 | * | 2.77 | *** | 0.23 |  | -1.64 | ** | -3.13 | * | 1.04 | * | -0.7 |  | 3.93 | *** | 0.47 |  |
| Age × SS_quadratic_ | 0.32 |  | -0.35 |  | 0.08 |  | -0.16 |  | -0.12 |  | -0.06 |  | 0.63 | * | -0.15 |  | 0.47 |  | -0.24 |  | 0.02 |  | -0.44 | * |
| Incentive | -2.49 |  |  |  | -2.71 |  |  |  | -1.09 |  |  |  | 8.28 | ** |  |  | 0.65 |  |  |  | 3.19 |  |  |  |
| Age × Incentive | -0.37 |  |  |  | -2.70 |  |  |  | -0.66 |  |  |  | -1.86 |  |  |  | -4.24 |  |  |  | -1.95 |  |  |  |
| SS_linear_ × Incentive | 1.11 | * |  |  | 0.55 |  |  |  | -0.86 |  |  |  | 0.90 |  |  |  | 0.65 |  |  |  | 1.53 | ** |  |  |
| SS_quadratic_ × Incentive | 0.24 |  |  |  | 0.20 |  |  |  | -0.01 |  |  |  | -0.54 |  |  |  | 0.03 |  |  |  | -0.04 |  |  |  |
| Age × SS_linear_ × Incentive | -0.28 |  |  |  | -1.59 | ** |  |  | 0.36 |  |  |  | -1.43 |  |  |  | -0.60 |  |  |  | -0.92 |  |  |  |
| Age × SS_quadratic_ × Incentive | 0.22 |  |  |  | -0.09 |  |  |  | 0.25 |  |  |  | 0.24 |  |  |  | 0.05 |  |  |  | 0.31 |  |  |  |

*** *p* < .001, ** *p* < .01, * *p* < .05, MLM: multilevel modeling, SS: Set size

Note. This study used reversed score for NASA-TLX Performance scale (0 = *failure*, 100 = *successful*): With the original score, the intercept is 25.8 and the beta values are the same values with the opposite sign.

Comparison with Hess et al. (2016): Differences in age comparisons between our study and that of Hess et al. (2016) should be qualified by noting that Hess's young adult sample was slightly older and likely represented a wider demographic range. Both studies recruited young adults ‘from the community’, but in Ann Arbor that community is for that age range dominated by students. Putting that aside, there also appear to be some differences in the older adult data between the studies, despite being quite similar on assessed demographics (age, education).

Our results, especially for the NASA-TLX Performance scale, differed somewhat from those of Hess et al. (2016). In terms of actual performance, while both of our age groups maintained near-ceiling performance through set size 4, with young adults continuing to do so even at set size 5, their older adults began showing declines at set size 2; young adults at set size 3. Our lowest group score was 15% at set size 9; both of their age groups had near-floor performance (<10% correct) by set size 8. For the NASA-TLX Performance subscale, while both studies had a significant Age × Set Size interaction, the nature of that interaction was quite different. In the present study, the self-ratings followed a similar pattern as actual performance – high at early set sizes, with the two age groups diverging around set size 4 and then re-converging at low self-ratings at set size 9. In Hess et al. (2016) the older adults rated their performance at low set sizes more poorly than did young adults, but the two age groups crossed at set size 6, so that subsequently young adults gave themselves poorer ratings than did older adults.

As with actual performance and the Performance subscale, our results for other subscales differed somewhat from those found by Hess and colleagues. For example, we did not find any age or Age × Set Size effects on Mental Demand, whereas they found that older adults gave higher ratings of Mental Demand at lower set sizes, with the two groups converging around set size 6. For Effort, Frustration, and Temporal Demand, where we found that older adults only gave higher ratings than young adults at the larger set sizes, they found either only a main effect of age (Effort and Temporal Demand; higher ratings for older adults), or a quadratic pattern (Frustration) with similar ratings for the two groups at the smallest and largest set sizes, but higher ratings for older adults at intermediate set sizes. One potentially interesting finding is that for Physical Demand, our control-condition results partially replicate theirs, with older adults showing a greater increase in ratings as set size increased, although we did not find a main effect of age, and older adults in the loss condition did not show this effect. We note this here as the strength of the age effect on Physical and Temporal Demand ratings was an unexpected finding in Hess et al. In our case, the unexpected component was the further interaction with Incentive. In general, the patterns suggest that our older adults in particular may have found the task easier than theirs at the lower set sizes, and only began to experience performance declines and corresponding dimensions of subjective demand at intermediate set sizes (5 or more items).

S4. State Attention and Motivation Questionnaire (SAMQ) all subscales


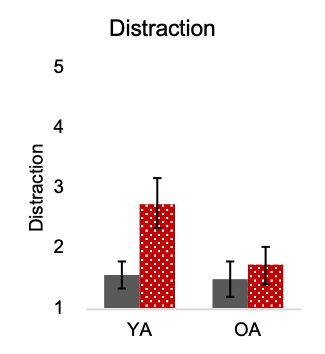


**


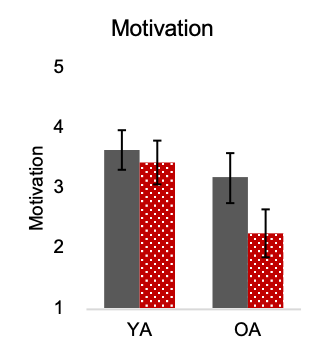


*

Different colors/pattern (control = black filled, loss = red dotted) are used to highlight the different conditions.YA: Younger adults, OA: Older adults, Error bars: 95% CI

Note. Q1. At times of this task, it was hard for me to keep my mind from wandering; Q2. During the task, my thoughts seldom drifted from the subject before me; Q3. I was easily bored during this task; Q4. I had difficulty in keeping my attention focused on this long tedious task; Q5. I found the possibility of [*Control*: getting feedback; *Loss*: losing money] to be distracting” as distraction; Q6. I found the possibility of losing money to be motivating. Participants responded with a scale from 1 (*definitely not true for me*) to 5 (*very true for me*).

S5. Intrinsic Motivation Inventory (IMI) results

|  | Interest/Enjoyment | | | Perceived Competence | | | Perceived Choice | | | Pressure/Tension | | |
| --- | --- | --- | --- | --- | --- | --- | --- | --- | --- | --- | --- | --- |
| Group | Mean (± *SD*) | | | Mean (± *SD*) | | | Mean (± *SD*) | | | Mean (± *SD*) | | |
| YA Control | 3.88 (± 1.34) | | | 3.71 (± 1.32) | | | 5.45 (± 0.98) | | | 3.72 (± 1.37) | | |
| YA Loss | 3.86 (± 1.15) | | | 3.79 (± 1.39) | | | 5.72 (± 0.90) | | | 4.34 (± 1.34) | | |
| OA Control | 4.26 (± 1.37) | | | 3.13 (± 1.27) | | | 5.72 (± 1.18) | | | 3.58 (± 1.54) | | |
| OA Loss | 4.55 (± 1.19) | | | 3.40 (± 1.11) | | | 5.84 (± 1.24) | | | 3.61 (± 1.49) | | |
| Effect | *F* | *p* | ŋ^2^_partial_ | *F* | *p* | ŋ^2^_partial_ | *F* | *p* | ŋ^2^_partial_ | *F* | *p* | ŋ^2^_partial_ |
| Age | 7.60 | 0.006 | 0.044 | 5.98 | 0.015 | 0.035 | 1.43 | 0.233 | 0.009 | 3.96 | 0.048 | 0.023 |
| Incentive | 0.46 | 0.499 | 0.003 | 0.82 | 0.366 | 0.005 | 1.39 | 0.240 | 0.008 | 2.20 | 0.140 | 0.013 |
| Age × Incentive | 0.64 | 0.427 | 0.004 | 0.26 | 0.613 | 0.002 | 0.21 | 0.647 | 0.001 | 1.77 | 0.185 | 0.011 |

YA: Younger adults, OA: Older adults

Comparison with Hess et al. (2016): We found that older adults gave higher scores on the Interest/Enjoyment scale than did young adults despite lower Competence scores. In contrast, Hess et al. (2016) did not find any age (or motivation instruction) effects on the IMI, again suggesting that there may be sampling differences between the studies.

S6. Meta-cognitive difference scores MLM results (*β*)

| Effect | Metacognition | |
| --- | --- | --- |
| Intercept | 8.40 | *** |
| Age | -2.50 |  |
| SS_linear_ | -0.37 |  |
| SS_quadratic_ | -0.47 | *** |
| SS_linear_ × Age | -1.67 | *** |
| SS_quadratic_ × Age | 0.31 |  |
| Incentive | -4.84 | * |
| Age × Incentive | 1.15 |  |
| SS_linear_ × Incentive | 0.12 |  |
| SS_quadratic_ × Incentive | 0.45 | * |
| Age × SS_linear_ × Incentive | 0.17 |  |
| Age × SS_quadratic_ × Incentive | -0.55 |  |

*** *p* < .001, ** *p* < .01, * *p* < .05, MLM: multilevel modeling, SS: Set size

S7. The full (Age × Incentive) *absolute* metacognitive accuracy graph

Different colors/lines (control = black solid line, loss = red dashed line) and shapes (triangle = young adults (YA), circle = older adults (OA)) are used to highlight the different conditions. SS: Set size, Error bars: 95% CI

S8. Excluded participants

| Reason for exclusion | Young adults (*n*) | Older adults (*n*) |
| --- | --- | --- |
| Did not pass the practice run (> 80% correct within 3 repeats) | 0 | 5 |
| Low MMSE (< 27) | n/a | 4 |
| Low ERVT (< 9) | 7 | 2 |
| Medical condition (ADHD) | 1 | 0 |
| Participant decided to drop out | 0 | 3 |

MMSE: Mini Mental State Examination score, ERVT: Extended Range Vocabulary Test score
